# Short-term study on risk-benefit outcomes of two spinal manipulative therapies in the treatment of acute radiculopathy caused by lumbar disc herniation: study protocol for a randomized controlled trial

**DOI:** 10.1186/s13063-015-0634-0

**Published:** 2015-03-27

**Authors:** Lei Han, Ping Zhao, Wei Guo, Jie Wei, Fei Wang, Yu Fan, Yi Li, Yaqing Min

**Affiliations:** PLA Spine Center of TCM Manipulative Orthopedics, Air Force General Hospital of PLA, No. 30, Fu Cheng Street, Hai Dian District Beijing, 100142 China

**Keywords:** Risk-benefit, Spinal manipulation, Mechanical intervention, Lumbar disc herniation, Acute radiculopathy, Randomized controlled trial

## Abstract

**Background:**

That patients with acute radiculopathy caused by lumbar disc herniation (LDH) will benefit from spinal manipulation (SM) treatment has been taken for granted, despite no solid evidence to support that claim. There is a demand for a win-win SM treatment that is both effective and less risky, and we attempt to use this trial to demonstrate such a treatment. In this study, Feng’s Spinal Manipulative Therapy (FSM) is selected as the observational SM. FSM can be performed with either manipulation or mobilization, and also can be easily mimicked as a sham SM.

**Methods/Design:**

Two hundred and sixteen qualified hospitalized participants will be randomly allocated to one of the three following groups: sham SM, mobilization, or manipulation, according to a ratio of 1:1:1. Participants in each group will receive specific FSM treatments four times, along with basic therapies over a course of 2 weeks. Two days after each SM appointment, risk outcomes will be assessed using a questionnaire developed to identify accompanying unpleasant reactions (AUR). The pain pressure threshold (PPT) will be measured paraspinally on the tender spot beside the involved joint before and immediately after each SM treatment. Relative risk (RR) of AUR, number needed to harm (NNH) and the 95% confidence intervals of each group will be calculated and compared. Benefit outcomes will be assessed by analyzing the following data recordings: the Numerical Rating Scale (NRS), Oswestry Disability Index (ODI), and Global Perceived Effect (GPE) before enrollment and at the 7th, and 15th day after the treatment. Analyses will include comparisons of NRS, ODI and changes at the different visit times among the three groups by Repeated Measures Data ANOVA, an evaluation of reduced scores of NRS and ODI after the therapy to determine if they meet the minimum acceptable outcome (MAO), and the determination of the minimal clinically important difference (MCID) by the average improvement in NRS and ODI scores of all participants who have been allocated to the category ‘improved’ on the GPE assessment.

**Trial registration:**

This trial is registered in Chinese Clinical Trial Register (ChiCTR) on 19 August 2013 (ChiCTR-TRC-13003496).

**Electronic supplementary material:**

The online version of this article (doi:10.1186/s13063-015-0634-0) contains supplementary material, which is available to authorized users.

## Background

Lumbar disc herniation (LDH) is one of most common diseases that produces low back pain and/or leg pain in adults, with an estimated annual incidence of 5 per 1,000 [1,2]. LDH patients suffer with acute or chronic pain, and even disabling pain in daily life, especially in the acute phase. The well-known pathomechanics of LDH is so-called radiculopathy originated from a protruded nucleus pulposus (PNP) breaking through the annulus fibrosis of a lumbar disc and causing an immunological irritation of neighboring nerve root(s) [3,4]. The segmental or regional biomechanical disturbances will be apparent in the lumbar spine. In addition to the neurological deficit of the involved nerve root, the mechanical disorder of the affected joint, known as ‘vertebral displacement’ [5] or ‘vertebral subluxation’ [6] is generally recognized, from the view of manual therapists, as another key point in the pathomechanics of LDH, which is deemed as a defensive reaction for joint protection that is induced by nerve root irritation caused by the PNP. Spinal manipulation (SM), therefore, could be reasonably applied to release the joint fixation of the involved segment [7] and to restore the flexibility of lumbar spine [8] in order to improve the biomechanical environment of the affected nerve roots, which is popularly recognized as the clinical reason for applying SM on LDH patients. On the other hand, it always has been challenging to apply this ancient skill of SM in a both safe and effective way on an LDH patient with acute radiculopathy because of the risk of producing harmful injury on the involved joints or/and disc. A recent systematic review concluded that SM is otherwise an alternative or complementary medical method, though with limited curative effect, and only better than a placebo if there is no severe adverse effect in its performance [9]. Another review indicated that some serious accidents occasionally happen during the performance of SM [10]. Despite a lack of consensus concerning its safety and effectiveness, SM is still broadly applied to LDH patients. Numerous doctors have achieved successful clinical experiences in SM practice for LDH patients and attempt to apply a sort of SM that will be win-win in that it is both effective and less risky. Many clinical reports [11-15] show that patients will benefit only if neither excessive nor insufficient stress is placed on involved joints in SM performance. The former will often cause harmful injury, and the later will be not powerful enough to reach the point of releasing joint fixation. In other words, an effective SM will load appropriate stress on the involved joints and produce non-noxious stimuli, while the harmful SM will overload torsion stress and produce noxious stimuli on the involved joints [16-18]. Based on an experiment, Cavanaugh JM [19] explained that if the tension of the joint capsule caused by the torsion force of the SM is increased to 44.2 ± 16.7% of the breaking threshold, the majority (83%) of non-nociceptors will exhibit saturation response, while with a tension increase of 47.2 ± 9.6% of the threshold, the nociceptors will result in a harmful sensation. It is of great clinical significance, therefore, to use an ‘appropriate mechanical intervention’ to achieve a balance between releasing the tension of the joint and reducing the risk of harmful injury during the SM performance.

The mechanics of current popular SM is believed to passively enforce a coupled motion and unlock a fixated joint in order to restore the range of motion (ROM) at the involved segment by torsion force. The motion can be regulated and directed by the therapist. The stress on the joint can be rapidly or slowly loaded according to different type of SM. The SM with the stress that is rapidly loaded is called ‘manipulation’ or ‘thrusting adjustment’ with a typical feature of high- velocity and low- amplitude (HVLA), while the SM with the stress that is slowly loaded is called ‘mobilization’ with a typical feature of low- velocity and variable- amplitude (LVVA). Hence, SM can be divided roughly into these two broad categories: ‘manipulation’ and ‘mobilization’. These two types of SM are believed to be the most popular SM nowadays [20,21]. It is understandable that these two types of SM have different kinematic and mechanical features of stress loading on involved joints during their performances. The type that is the ‘appropriate mechanical intervention’ has not yet been proven, although the clinical significance of both is widely recognized. There has been high demand for an objective evaluation on the risk-benefit outcomes of these two types of SM.

### Aims

The aims of this study are as follows:

1. To evaluate the short-term outcomes of risk-benefit from two types of SM, known as ‘manipulation’ and ‘mobilization’ in comparison to a sham SM for the treatment of LDH patients with acute radiculopathy.
2. To understand the best ‘appropriate mechanical intervention’ of two types of SM in treating LDH patients with acute radiculopathy.

## Methods/Design

### Study design

This is a prospective, randomized, parallel, placebo-controlled study of the short-term risk-benefit outcomes of two types of SM in a treatment of LDH patients with acute radiculopathy. A placebo group of patients treated by sham SM is specially designed along with the two study groups andwill present the natural progression of an LDH patient under baseline treatment with conventional treatments. After signing the informed consent, the patients enrolled in the study will undergo a 2-weeks course of treatment.

### Participants

The participants, aged 24 to 45 years, will be consecutively selected from the hospitalized LDH patients in the PLA Spine Center of Manipulative Orthopedics at PLA Air Force General Hospital in Beijing. All qualified candidates must meet the diagnostic criteria of LDH [22] for inclusion, and those who are not suitable for the study will be excluded (see Table 1). The participants will be guided by nurses to fill out demographic data forms after enrollment, thereby providing information on age, sex, height, weight, waistline, *etcetera,* and to sign the agreement. One doctor appointed by the research team, who is blinded to the grouping, will undertake physical examinations on all participants and record data from his findings. In addition to quantifying and recording the results of his physical exam, he will assess and record the participant according to the Numerical Rating Scale (NRS) and the Oswestry Disability Index (ODI). In addition, the participants will also respond to the Minimum Acceptable Outcome Questionnaire (MAO), including the minimum expectations for NRS and ODI after treatment.

**Table 1:** **Inclusion and exclusion criteria**

**Inclusion criteria**	**Exclusion criteria**
• Low back pain, lower limb pain is radiatingly distributed and correlates with the involved PNP as proven by MRI	• Congenital abnormalities, such as lumbosacral vertebrae crack, spondylolysis and transitional vertebrae, *etcetera*
• Two of four possible signs of neurological disorders: muscle atrophy, weakness, paresthesia and change in reflection that appeared in the nerve distribution areas	• Injury, such as fracture
	• Inflammatory and metabolic diseases, such as tuberculosis, ankylosing spondylitis, or osteoporosis
• Either straight leg raising test or femoral nerve traction test is positive	• Degenerative diseases, such as degenerative lumbar spondylolisthesis, degenerative lumbar instability, lumbar degenerative scoliosis and lumbar spinal stenosis, *etcetera*
• EMG or NCV shows nerve root injury correlated with involved segment	
• MRI image reveals a PNP of a single lumbar segment in accordance with the nerve root irritation signs detected by the examiner	• Vascular and visceral reflex low back pain, such as digestive system diseases, gynecological diseases, or abdominal aortic aneurysm
• Tenderness, swelling and thickness of supraspinous ligament could be palpated on the involved segment. Regional and/or radiating tenderness could be irritated by paraspinal palpation	• Spinal tumors from inside and outside spinal canal, such as large spinal arachnoid cysts, or diabetic peripheral neuropathy
	• Patients receiving oral medication, physical therapy and other modality
• Participants suffered with acute pain no more than 6 weeks	• Patients with foot-drop caused by peroneal nerve paralysis
• NRS score is more than or equal to 6 and ODI score is more than or equal to 40	• Patients with saddle anesthesia or defecation dysfunction caused by CES
• Participants must have no prior experience of being treated by FSM (see below)	• Patients with depression, anxiety and other mental disorders
• Participants must sign the informed consent	• Patients with open lumbar surgeries

CES, cauda equina syndrome; EMG, electromyogram; FSM, Feng’s Spinal Manipulative Therapy; MRI, magnetic resonance imaging; NCV, nerve conduction velocity; NRS, numerical rating scale; ODI, Oswestry Disability Index; PNP, protruded nucleus pulposus.

Random allocationA total of 216 cases of qualified participants will be enrolled in the study. A doctor from the research team is designated to randomly allocate participants to the groups. According to a ratio of 1:1:1, the doctor will assign the participants to one of the three following groups: group 1 for placebo (sham SM), group 2 for SM with mobilization, or group 3 for SM with manipulation. The assignment to groups is based on the random number sequence generated by SPSS 19.0 statistical software. The group information will be written on a card and sealed in an envelope. The determination of grouping will not be disclosed until the envelope is opened by the designated doctor when he encounters a qualified participant.

Due to ethical reasons, if patients in any groups have not realized their expected result from the treatment at the end of two weeks observation, they may ask their doctors to modify the treatments based on their own choices. Any participant who discontinues treatment during the study period also may be followed up so that the reason for discontinuation can be recorded.

### Intervention

All participants in the three groups will be given basic intervention in addition to thespecial interventions of SM that will be applied to the different treatment groups. The basic intervention will start from the first day after enrollment in the study. The SM intervention will be applied four times: on the 1^st^, 4^th^, 8^th^ and 11^th^ days after enrollment.

### Basic therapies

Due to ethical reasons, some basic conventional treatments have to be applied for all participants in the three groups (see Table 2).

**Table 2:** **Basic therapies**

**Basic therapies**	**Direction for use**
Bed rest	Stay in bed for 18 to 20 hours per day including sleeping time.
Dehydration	20% Mannitol injection, 250 ml, VD within 30 min, once a day for the first 5 days.
NSAID	After each SM therapy, the patient will be given 500 mg Paracetamol orally for 2 days (b.i.d.) in order to reduce the adverse regional irritation caused by SM.
Regional fomentation	Low back fomentation is applied with a hot pack of Chinese herbs* for 20 minutes, twice per day during the observation period.
Walking exercise	Patient could take a walking exercise with the assistance of a wheeled walker aid if no serious pain has been induced. The walking time should be limited to less than 20 minutes each time and to three times a day.

NSAID, nonsteroidal anti-inflammatory drug; SM, spinal manipulation.

*The hot pack is filled with more than ten types of Chinese herbs and is produced by the hospital. It has been successfully applied for LDH patients in the hospital for more than 40 years.

### Spinal manipulation

**Figure 1:**
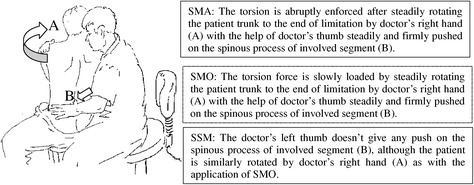
**Chart of Feng’s Spinal Manipulative Therapy (FSM).** See the difference among the spinal manipulation (SMA), spinal mobilization (SMO) and sham spinal manipulation (SSM).

In this study, Feng’s Spinal Manipulative Therapy (FSM) [23] is selected as the observational SM to be studied, which is a very popular Chinese SM and created by Dr. Tian-you Feng in the 1970s. The FSM technique is performed while the patient is in a sitting position, and the involved segment is precisely pressed by a gentle but stabilized torsion force. Based on FSM treatment, Dr. Feng created ‘the PLA Spine Center of Manipulative Orthopedics’ in the 1980s, and now the center is very famous for Spinal Manipulative Therapy and attracts thousands of patients from throughout China and abroad, which gave us the opportunity to design this trial.. The lumbar concept of the FSM technique is basically similar to any type of traditional spinal manipulation with a focus on loading a torsion force at the involved joints, except more gently and delicately, usually without the signature cracking sound. Only two rotation movements to each side and within the range of motion (ROM) of the lumbar spine were applied during a single treatment (see Figure 1).

The torsion stress can be quickly loaded on the involved joint during the rotation, such as in HVLA manipulation, with the help of doctor’s thumb steadily and firmly pushed against the spinous process of the involved segment until the end of the ROM or until pain is induced during the process. Torsion stress can also be slowly loaded in FSM performance following the principle of mobilization in SM. The former is applied in one group for spinal manipulation (SMA), and the latter is applied in another group for spinal mobilization (SMO). Both SMA and SMO are clinically and effectively applied in subjects who are hospitalized for acute radiculopathy caused by LDH.

The FSM can be easily mimicked by the sham SM (SSM) and applied to the patients in the placebo-control group without their consciousness if they have no experience of FSM treatment before. It is especially difficult to distinguish between SMO and SSM. The only difference between them is the regional stress at the involved segment given by doctor’s thumb push during the rotation, which plays a key role in directing the torsion force on the involved segment. On the other hand, there is no signature thrusting approach with a cracking sound of the joints to demonstrate completeness in SMO, which will be an easy disguise for SSM intervention. The patients in the SSM group, therefore, will be blind to whether they have received SMO or SSM treatment when they are hospitalized together. The different specific procedures of the FSM performance in three groups for SSM, SMO, and SMA are shown in Table 3.

**Table 3:** **Different specific procedures of Feng’s Spinal Manipulative Therapy (FSM) performance in three groups**

**Three types of FSM**	**Different specific procedures**
SSM (placebo)	No further torsion stress loaded on the involved joint after trunk rotation is ended at the limit of ROM, although the doctor’s thumb is gently located on the lower part of the lumbar spine.
SMO (mobilization)	After trunk rotation reaches the end of the ROM of the lumbar spine, more torsion stress is gently and slowly loaded on the patient’s shoulder and the involved joint during the rotation within 3 seconds with the feature of LVVA with the help of the doctor’s thumb steadily and firmly pressed against the spinous process of the involved segment accompanied by no cracking sound most of the time.
SMA (manipulation)	After trunk rotation reaches the end of the ROM of the lumbar spine, more torsion stress is forcefully and quickly loaded on the patient shoulder and the involved joint within 0.5 seconds with the feature of HVLA with the help of the doctor’s thumb swiftly and firmly pressed against the spinous process of the involved segment accompanied by cracking sound some of the time.

FSM, Feng’s Spinal Manipulative Therapy; HVLA, high velocity and low amplitude; LVVA, low velocity and variable amplitude; ROM, range of motion; SMA, spinal manipulation; SMO, spinal mobilization; SSM, sham spinal manipulation.

### Withdrawing from the study

Although FSM has proven to be one of the most effective SMs and with less adverse effects [24], it will, nevertheless, produce an occasional adverse effect, as with any SM. These adverse events will be classified according to the following:

1. Severe adverse event. Though rare, these situations are believed to be emergency situations, for example, the radicular pain intensity is rated above 8/10 on an 11-point NRS, ankle-foot sensorimotor function is suddenly absent, or defecation dysfunction and saddle anesthesia occur, all of which have been defined as a serious adverse event. If a participant reports a severe adverse event to their therapist or to the research staff, they will be withdrawn from the study and referred to their principal investigator for further emergency treatment depending on the nature of the event.
2. Moderate adverse event. If the participants feel their original pain increased regionally or/and radically after the SM intervention, with its intensity rated below 8/10 on an 11-point NRS, 500 mg of Paracetamol will be given orally b.i.d., increasing to t.i.d. for two days in order to reduce the adverse irritation. The dose will be recorded and included in the final analysis.

### Ethical approval

All participants will provide voluntary written informed consent with a full understanding of what study participation entails and the potential risks. Ethics approval has been obtained from Chinese Ethics Committee of Registering Clinical Trials (Reference number: ChiECRCT-2013016).

### Risk-benefit outcomes

The latest OUCH study [25] concluded that a substantial proportion of adverse events after chiropractic treatment may result in nonspecific effects. In this study, the purpose of a comprehensive analysis of different manipulations is aimed at the risk-benefit outcomes involved in each manipulative treatment, not a comparison between them in terms of efficacy and safety.

### Risk outcomes

Accompanying Unpleasant Reactions (AUR): The AUR questionnaire is based on clinical reports from Bruce F. Walker [25], E Ernst [10], and Drew Oliphant [26], with some reference to the authors’ clinical experience, and some other related references [27,28]. The AUR questionnaire [see Additional file 1] is mainly composed of two parts, including the anticipated part (AAUR) and unanticipated part (UAUR), which will be completed two days after each SM treatment and returned in a reply envelope. The AAUR consists of increased pain regionally and/or radiatingly, increased numbness or weakness in the leg and/or foot, paraspinal muscle stiffness and other items. Each item is graded for three aspects including start-end time (sustained response), degree of severity (graded according to three levels as mild, moderate or severe), and disturbance in daily life activities (sitting, lying, standing, walking, *etcetera*); meanwhile, the UAUR consists of saddle anesthesia, or defecation dysfunction caused by Cauda Equina Syndrome (CES), severely radicular pain, or by the sudden absence of ankle-foot sensorimotor function, caused by the herniated nucleus pulposus increasing or rupturing.

Pain Pressure Threshold (PPT): The PPT is measured by a PPT detector (FlexiForce’s Economical Load & Force System, ELF) paraspinally on the tender spot beside the involved joint before and immediately after each SM treatment (assessed a total of eight times). The head of the ELF detector is pressed on the spot with a sustained increasing stress at a speed of 0.5 kg/cm ^2^/s until the patient feels pain, which is recorded as the starting PPT. Furthermore, the pressure continues to increase until an unbearable dodge response is evoked in the patient, which is recorded as the peak PPT.

### Benefit outcomes

All participants must be examined and checked using the indexes once they are hospitalized (Visit 1) and then rechecked at the 7^th^ (Visit 2) and 15^th^ (Visit 3) day after the treatment.

Numerical Rating Scale (NRS): The NRS contains an 11-point scale varying from 0 (no pain) to 10 (worst pain imaginable) [29]. It is a valid, reliable and responsive measure of pain intensity [30,31].

Oswestry Disability Index (ODI): The ODI assesses the impacts of low back pain or leg pain on the physical function and activities of daily living [32]. It has been shown to have high levels of reliability, validity and responsiveness in patients with low back pain [33,34].

Global Perceived Effect (GPE): The GPE contains a 6-point scale varying from 1 (completely recovered) to 6 (much worse) [35]. It can be clustered in three main categories: ‘improved’, ‘unchanged’, and ‘deteriorated’ based on previous studies [36,37]. The category ‘improved’ included patients who scored ‘1’ or ‘2’ on the GPE. Patients who scored ‘3’, ‘4’, or ‘5’ were categorized as ‘unchanged’. The category ‘deteriorated’ included patients who scored ‘6’ on the GPE.

### Statistical analysis

The statistical analysis is designed by a specialist from the Institute of Basic Clinical Research, China Academy of Chinese Medical Science. Data will be analyzed by statistic software (SPSS Version 19.0) in this study.

### Risk analysis

The AUR frequency and severity of each group will be calculated. The Relative Risk (RR) [38] of AUR and the 95% confidence intervals of each group will be calculated and compared. The Number Needed to Harm (NNH) [39] will be calculated with 95% confidence intervals to identify the number of individuals needed to be exposed to an SM treatment over a specific period to cause harm in one patient that would not otherwise have been harmed.

Furthermore, correlations between the PPT changes and the AUR will be analyzed by linear regression to explore the relationship between them. The relevance might be helpful in predicting AUR during the treatment. The primary safety analysis will be based on the Safety Set (SS), which was defined as the data from the subjects who were randomized into groups and received at least one treatment, and had at least one safety assessment after treatment.

### Benefit analysis

First, the NRS, ODI and changes at different visit times (Visit 1, 2 and 3) among the three groups will be analyzed and compared by Repeated Measures Data ANOVA.

Second, the reduced scores of NRS and ODI after the therapy will be evaluated to find out if they meet the minimum acceptable outcome (MAO) [40] based on the data collected at the first sample visit (Visit 1). The matching cases will be calculated, and the differences between the groups will also be compared by Chi-square test.

Third, the improved scores of NRS and ODI at the terminal visit (Visit 3) compared with that of first visit (Visit 1) will be evaluated. The Minimal Clinically Important Difference (MCID) [41] will be determined by the average improvement in the scores of all participants who are allocated as the category ‘improved’ on the GPE assessment.

The data analyzed for benefit outcome are based on a Full Analysis Set (FAS), which is defined as the data collected in the first visit (Visit 1) from all randomized subjects and at least one post-treatment assessment (Visit 2 or 3). In addition, Intention ToTreat Analysis (ITT) will be performed.

### Blinding

As we all fully understand, it will not be possible to blind the therapists, as this is an RCT design for a clinical study on manual therapy. Therefore, we must apply a third party assessment with regard to objective evaluation. The statistician and data collecting doctors will be blinded to the grouping of the samples. Although the patients are suppose to be blinded to their treatment and grouping, we still will verify that they remain blinded by assessing them with a Bang Index (BI) questionnaire just after the observation. The Bang Index (BI) [42] will help us to determine if a significant proportion of the participants guessed that they were in a different treatment group. A BI value of < −0.2 indicates that a significant proportion guessed wrongly, a BI between 0.2 and −0.2 indicates that participants guessed randomly, and a BI >0.2 indicates that the study is unblinded.

### Sample size

A pilot randomized trial already has been conducted with ten participants in each group. At the end of 2-weeks treatment, the NRS differences (standard deviation, S.D.) in the SMA and SMO groups were 1.5 (2.5) and 1.8 (2.8) respectively, compared to the SSM group. Based upon the selected former group data, a power analysis (power = 90%, alpha = 0.05) indicated that 180 participants (60 per group) would be needed to complete an adequately powered randomized controlled trial. Considering a 20% dropout rate, a reasonable sample size will be 216 cases with at least 72 cases in each group.

### Study flow

**Figure 2:**
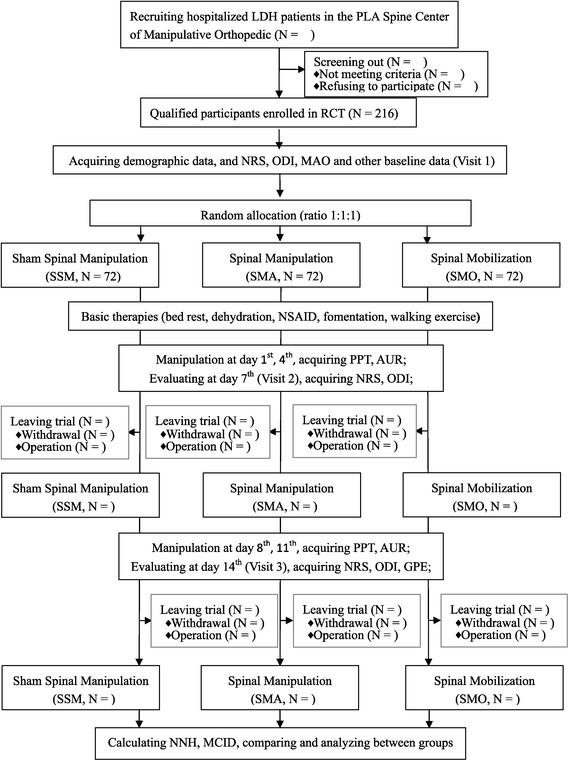
**Flow chart of the study.**

A flow chart of the study is seen in Figure 2.

## Discussion

The key factor affecting the quality of this clinical trial is the quality control of the manipulative intervention given by the manipulative specialist. It is understandable that the operational difference might be produced by different therapists, though they may perform a same technique of SM. It will be avoided to some extent in this study by the following:

1. Only three senior doctors in the center will be appointed to perform the SM in the study. The doctors need to be very experienced in the SM performed. Even so, they will not perform the treatment until they have been trained for the different specific requirements of SM in the different study groups.
2. Before the study, a quantitative stress-testing tool called ‘ELF’ will be applied in the training program for producing a standardized rotating stress on the involved segment during the SM process in different groups.
3. The same participant will be treated by the same physician during the period of treatment, because changes of physician will give him or her negative psychological feelings. Through the above measures, homogeneity of SM could be achieved, and it was ensured that SM is carried out according to different specific operating procedures for the enrolled participants.

It always has been difficult to set up a placebo control in a comparative clinical study for manual therapy. SSM as a placebo control would not be blinded to the subjects and might give him a negative psychological influence. In this study, FSM is selected as the intervention of SM, which is easily mimicked as SSM and can be applied to the participants in the placebo-control group without their recognition if they have no prior experience of FSM treatment. The only difference between SMO and SSM is the regional stress at the involved segment given by doctor’s thumb push during the rotation, which plays a key role in directing the torsion force on the involved segment. On the other hand, there is no signature thrusting approach with cracking sound of the joints to demonstrate completeness in either the SMO or the SSM treatment. The patients in either SSM or SMO groups, therefore,will be blinded to whether they had received SM treatment or not. SMA treatment certainly will be taken for granted since it is similar to the subjects’ expectation of SM therapy. It is, therefore, extremely important to emphasize in this study that the subjects selected have no prior experience of FSM treatment.

Subject compliance is another problem difficult to resolve in this kind of clinical observation when different types of SM therapies are employed in the same environment. In order to solve the problem in this study, FSM was selected as the observational SM to be studied. The FSM is very effective, has been popular in mainland China for more than 40 years and is officially recognized by China’s health care administration, which is significantly helpful in improving patient compliance in the study. Both SMO and SMA are generally applied in the center where the subjects are hospitalized. The SSM mimics the SMO and is, therefore, easily disguised to the participants. This will make the participants believe that they are treated with one of the best cares of manual therapy during the observational period.

If a subject in the SSM group becomes aware, by accident, that his/her treatment is a sham SM, his compliance to the study possibly will be affected. The participant will be immediately advised to accept FSM treatment along with the others in the center and will be listed as a dropout sample. If the participants in any observational SM group are unsatisfied with the SM treatment received, he/she will also be ruled out from the study in order not to affect results of the study by emotional resistance. It is well known that some patients may suffer from a short-term adverse effect of increasing pain after the SM treatment, which likely will be comprehensively accepted by the participants when informed in advance. Those who have strong resistance to the adverse effect by SM will be ruled out from the study. During the observation, if the subject cannot receive an SM treatment in time due to special reasons, a postponed SM treatment is allowed, but the delay can be no more than 2 days. Because there is a possibility that the subject may violate the treatment protocol by taking other prohibited interventions, such as additional nonsteroidal anti-inflammatory drugs, a cleansing period of this additional medication or treatment, therefore, should be added, but no more than 3 days. Any accidental interference over the patient compliance should be recorded in detail and the corresponding evaluation will be processed at the final analysis.

## Trial status

The first participant was allocated on 30 January 2014, and the final participant is anticipated to be allocated in July 2015.

## Additional file

Additional file 1:
**Accompanying Unpleasant Reactions (AUR) Questionnaire.** Survey instrument developed for this study to gather information about unpleasant reactions accompanying with spinal manipulation treatment.

## Abbreviations

AUR: accompanying unpleasant reactions
FSM: Feng’s Spinal Manipulative Therapy
GPE: global perceived effect
HVLA: high velocity and low amplitude
LDH: lumbar disc herniation
LVVA: low velocity and variable amplitude
MAO: minimum acceptable outcome
MCID: minimal clinically important difference
NRS: numerical rating scale
ODI: Oswestry Disability Index
PNP: protruded nucleus pulposus
PPT: pain pressure threshold
RR: relative risk
NNH: number needed to harm
SMA: spinal manipulation
SMO: spinal mobilization
SSM: sham spinal manipulation
