# Supplementary material for: Short-term study on risk-benefit outcomes of two spinal manipulative therapies in the treatment of acute radiculopathy caused by lumbar disc herniation: study protocol for a randomized controlled trial
Source: Trials. 2015 Mar 27;16:122. doi: 10.1186/s13063-015-0634-0 (PMC4380109; doi:10.1186/s13063-015-0634-0)
Supplement: Additional file 1: — Accompanying Unpleasant Reactions (AUR) Questionnaire. Survey instrument developed for this study to gather information about unpleasant reactions accompanying with spinal manipulation treatment. [file 13063_2015_634_MOESM1_ESM.pdf]

Accompanying Unpleasant Reactions Questionnaire

Apart from measuring any improvement of your condition we are seeking to develop an understanding of unpleasant reactions that people may experience accompanying with spinal manipulation treatment. Unpleasant reactions in this study may include anticipated reactions such as pain, numbness or muscle tightness, and some other unanticipated events. Your support in completing this questionnaire would be greatly appreciated as it will provide valuable information about the types of adverse events people may experience accompanying with spinal manipulation treatment. We estimate that this questionnaire will take 10-15 minutes to complete. Thank you for taking the time to complete this questionnaire.

Part A

Question 1. Did you experience any new unwelcome symptoms or an increase of your presenting symptoms during the first 48 hours (two days) after treatment? This includes pain, numbness, tightness or restrictions in daily activities

❑ Yes ❑ No

If you answered **No** to **Question 1**, please stop here. If you answered **Yes** to **Question 1**, please continue

Part B

If you experienced any complaints after spinal manipulation treatment, please mark the boxes and scales below to indicate how long the complaint started, how long the complaint lasted, and how severe the complaint was.

Question 2. Did you feel any lumbar muscle tightness or stiffness after spinal manipulation appointment

❑ Yes ❑ No

If yes, please answer the next three questions.

How long after the appointment did you notice the stiffness?

❑ less than 10 minutes ❑10 minutes to 1 hour ❑ 1 to 4 hours ❑ 4 to 24 hours ❑ more than 24 hours

How long did the stiffness last for?

❑ less than 1 hour ❑ 1 to 12 hours ❑ 12 to 24 hours ❑24 to 48 hours ❑ more than two days

Please indicate how severe the stiffness was (1-3=mild; 4-6=moderate; 7-10 severe)

0 1 2 3 4 5 6 7 8 9 10

❑ Yes ❑ No

If **Yes** , please continue

Please indicate how severe the stiffness was before the appointment if it existed

0 1 2 3 4 5 6 7 8 9 10

Question 3. Did you feel increased pain on waist, hip or leg, foot regionally after spinal

manipulation appointment

❑ Yes ❑ No

If yes, please answer the next three questions.

How long after the appointment did you notice the increased pain?

❑ less than 10 minutes ❑10 minutes to 1 hour ❑ 1 to 4 hours ❑ 4 to 24 hours ❑ more than 24 hours

How long did the increased pain last for?

❑ less than 1 hour ❑ 1 to 12 hours ❑ 12 to 24 hours ❑24 to 48 hours ❑ more than two days

Please indicate how severe the pain was (1-3=mild; 4-6=moderate; 7-10 severe)

0 1 2 3 4 5 6 7 8 9 10

❑ Yes ❑ No

If **Yes** , please continue

Please indicate how severe the pain was before the appointment if it existed

0 1 2 3 4 5 6 7 8 9 10

Question 4. Did you feel increased radiating pain on hip, leg, or foot after spinal manipulation appointment

❑ Yes ❑ No

If yes, please answer the next three questions.

How long after the appointment did you notice the increased radiating pain?

❑ less than 10 minutes ❑10 minutes to 1 hour ❑ 1 to 4 hours ❑ 4 to 24 hours ❑ more than 24 hours

How long did the increased radiating pain last for?

❑ less than 1 hour ❑ 1 to 12 hours ❑ 12 to 24 hours ❑24 to 48 hours ❑ more than two days

Please indicate how severe the radiating pain was (1-3=mild; 4-6=moderate; 7-10 severe)

0 1 2 3 4 5 6 7 8 9 10

❑ Yes ❑ No

If **Yes** , please continue

Please indicate how severe the radiating pain was before the appointment if it existed

0 1 2 3 4 5 6 7 8 9 10

Question 5. Did you feel increased numbness or weakness on leg or foot after spinal manipulation appointment

❑ Yes ❑ No

If yes, please answer the next three questions.

How long after the appointment did you notice the increased numbness or weakness?

❑ less than 10 minutes ❑10 minutes to 1 hour ❑ 1 to 4 hours ❑ 4 to 24 hours ❑ more than 24 hours

How long did the increased numbness or weakness last for?

❑ less than 1 hour ❑ 1 to 12 hours ❑ 12 to 24 hours ❑24 to 48 hours ❑ more than two days

Please indicate how severe the numbness or weakness was (1-3=mild; 4-6=moderate; 7-10 severe)

0 1 2 3 4 5 6 7 8 9 10

❑ Yes ❑ No

If **Yes** , please continue

Please indicate how severe the numbness or weakness was before the appointment if it existed

0 1 2 3 4 5 6 7 8 9 10

Question 6. Did you experience any other unpleasant symptoms not listed in the above after spinal manipulation appointment

❑ Yes ❑ No

If yes, please What fill directly in the following and answer the next three questions.

What kind of discomfort ⅰ**:** ____________________________

How long after the appointment did you notice the discomfort?

❑ less than 10 minutes ❑10 minutes to 1 hour ❑ 1 to 4 hours ❑ 4 to 24 hours ❑ more than 24 hours

How long did the discomfort last for?

❑ less than 1 hour ❑ 1 to 12 hours ❑ 12 to 24 hours ❑24 to 48 hours ❑ more than two days

Please indicate how severe the discomfort was (1-3=mild; 4-6=moderate; 7-10 severe)

0 1 2 3 4 5 6 7 8 9 10

❑ Yes ❑ No

If **Yes** , please continue

Please indicate how severe the discomfort was before the appointment if it existed

0 1 2 3 4 5 6 7 8 9 10

What kind of discomfort ⅱ**:** ____________________________

How long after the appointment did you notice the discomfort?

❑ less than 10 minutes ❑10 minutes to 1 hour ❑ 1 to 4 hours ❑ 4 to 24 hours ❑ more than 24 hours

How long did the discomfort last for?

❑ less than 1 hour ❑ 1 to 12 hours ❑ 12 to 24 hours ❑24 to 48 hours ❑ more than two days

Please indicate how severe the discomfort was (1-3=mild; 4-6=moderate; 7-10 severe)

0 1 2 3 4 5 6 7 8 9 10

❑ Yes ❑ No

If **Yes** , please continue

Please indicate how severe the discomfort was before the appointment if it existed

0 1 2 3 4 5 6 7 8 9 10

What kind of discomfort iii: ______________________

How long after the appointment did you notice the discomfort?

❑ less than 10 minutes ❑10 minutes to 1 hour ❑ 1 to 4 hours ❑ 4 to 24 hours ❑ more than 24 hours

How long did the discomfort last for?

❑ less than 1 hour ❑ 1 to 12 hours ❑ 12 to 24 hours ❑24 to 48 hours ❑ more than two days

Please indicate how severe the discomfort was (1-3=mild; 4-6=moderate; 7-10 severe)

0 1 2 3 4 5 6 7 8 9 10

❑ Yes ❑ No

If **Yes** , please continue

Please indicate how severe the discomfort was before the appointment if it existed

0 1 2 3 4 5 6 7 8 9 10

Part C

If you had any additional problems or increasing difficulties with the activities of daily living after spinal manipulation appointment

❑ Yes ❑ No

if yes, please indicate

Question 7. Did you have more difficulty with getting up, or maintaining posture, or changing posture in bed

❑ Yes ❑ No

Question 8. Did you have more difficulty with sitting , or rising from a chair

❑ Yes ❑ No

Question 9. Did you have more difficulty with standing

❑ Yes ❑ No

Question 10. Did you have more difficulty with walking

❑ Yes ❑ No

Question 11. Did you have more difficulty with dressing such as wearing trousers, socks, shoes

❑ Yes ❑ No

Question 12. Did you have more difficulty with personal hygiene such as washing your face, or brushing your teeth

❑ Yes ❑ No

Question 13. Did you have more difficulty with sleep disorders such as falling asleep with difficulty, or waking up easily because of pain

❑ Yes ❑ No

Question 14. Did you have more difficulty with defecating because of pain

❑ Yes ❑ No

If there were any other problems you experienced after spinal manipulation appointment, please list on the space below

____

____

____

Part D

If you experienced severe adverse reactions after spinal manipulation treatment, please sign in the following questions

Question 15. Did you experience saddle anesthesia, or defecation dysfunction

❑ Yes ❑ No

Question 16. Did you experience severely radicular pain, or ankle-foot sensorimotor function suddenly missing

❑ Yes ❑ No

Part E

Question 17. Did you have any additional therapy apart from doctor's treatment for your complaints during the two weeks of spinal manipulation treatment

❑ Yes ❑ No

Thank you for taking the time to complete this questionnaire
